# Fragrance components in cosmetic products: an analysis of labels^؆^

**DOI:** 10.1016/j.abd.2026.501325

**Published:** 2026-03-27

**Authors:** Ana Carolina Rodrigues, Mariana de Figueiredo Silva Hafner, Rosana Lazzarini

**Affiliations:** a Dermatology Clinic, Irmandade da Santa Casa de Misericórdia de São Paulo, São Paulo, SP, Brazil; b Faculty of Medical Sciences, Santa Casa de São Paulo, São Paulo, SP, Brazil

Dear Editor,

Fragrances are chemical compounds, of natural or synthetic origin, incorporated into formulations aiming at providing a pleasant aroma or masking undesirable odors.^1^ They play a significant role in the sensory appeal of various products, especially cosmetics, contributing significantly to the consumer’s experience. Several chemical substances are used as fragrances in the production of perfumes and aromas used by the cosmetic industry. However, these compounds are among the most frequent causes of Allergic Contact Dermatitis (ACD).^2^, ^3^

Despite the existence of national and international regulations that guide the labeling of fragrances in cosmetics, previous studies indicate that a significant portion of individuals affected by ACD report difficulty in understanding the ingredients listed on the labels, which follow the International Nomenclature of Cosmetic Ingredients (INCI). This limitation compromises the identification and consequent avoidance of the allergens involved.^4^

Given this context, the present study aimed to evaluate the presence of the main fragrances in a sample of cosmetic products available on the Brazilian market. A total of 501 labels of cosmetics sold in the city of São Paulo were analyzed, which were categorized as shown in Table 1. The analysis consisted of verifying the presence of the 26 fragrances listed in Collegiate Board Resolution (RDC) N. 530, of August 4, 2021, of the National Health Surveillance Agency (ANVISA)^5^ (Table 2). According to this regulation, such substances must be declared on the labeling of personal hygiene products, cosmetics and perfumes when present at concentrations greater than 0.001% in leave-on products and 0.01% in rinse-off products.

**Table 1:** Analyzed cosmetic products, grouped by category.

Categories	Products
Rinse-off hair care (63 products)	Shampoo, conditioner, hair moisturizer, hair mask, hair ampoule
Leave-on hair care (38 products)	Styling cream, texturizing cream, finishing gel, hairsprays, leave-in conditioner, curl-defining serum, hair oil, curl definer.
Hair dye (51 products)	Hair dyes, toners, hair makeup
Rinse-off facial care (52 products)	Cleansing gel, facial soap, facial scrub, facial cleansing mousse, shaving cream
Leave-on facial care (45 products)	Hyaluronic acid, micellar water, anti-aging cream, facial moisturizer, makeup, makeup remover, multi-purpose lotion, facial rejuvenator, daily revitalizer, serum, vitamin C, facial toner, facial mask, aftershave lotion
Rinse-off body care (60 products)	Body scrub, shower gel, in-shower moisturizer, bath oils, soaps
Leave-on body care (85 products)	Deodorant, antiperspirant, alcohol, moisturizers, emollient butter, body deodorant, perfumed lotion, scar reduction gel, talcum powder.
Sunscreen (56 products)	Sunscreen with and without color.
Toothpaste (39 products)	
Alcohol gel (12 products)

**Table 2:** List of fragrance and aroma components that must be indicated on the labels of personal hygiene products, cosmetics and perfumes according to ANVISA regulations.

Substance	INCI
2-(4-tert-Butylbenzyl) propionaldehyde (CAS 80-54-6)	*Butylphenyl methylpropional*
3-Methyl-4-(2,6,6-trimethyl-2-cyclohexen-1-yl)-3-buten-2-one (CAS 127-51-5)	*Alpha-isomethyl ionone*
Amyl cinnamal (CAS 122-40-7)	*Amyl cinnamal*
Amylcinnamyl alcohol (CAS 101-85-9)	*Amylcinnamyl alcohol*
Anisyl alcohol (CAS 105-13-5)	*Anise alcohol*
Benzyl alcohol (CAS 100-51-6)	*Benzyl alcohol*
Benzyl benzoate (CAS 120-51-4)	*Benzyl benzoate*
Benzyl cinnamate (CAS 103-41-3)	*Benzyl cinnamate*
Benzyl salicylate (CAS 118-58-1)	*Benzyl salicylate*
Cinnamal (CAS 104-55-2)	*Cinnamal*
Cinnamyl alcohol (CAS 104-54-1)	*Cinnamyl alcohol*
Citral (CAS 5392-40-5)	*Citral*
Citronellol (CAS 106-22-9)	*Citronellol*
Coumarin (CAS 91-64-5)	*Coumarin*
d-Limonene (CAS 5989-27-5)	*D-LIMONENE*
Eugenol (CAS 97-53-0)	*Eugenol*
Farnesol (CAS 4602-84-0)	*Farnesol*
Geraniol (CAS 106-24-1)	*Geraniol*
Hexyl cinnamaldehyde (CAS 101-86-0)	*Hexyl cinnamal*
Hydroxy-citronellal (CAS 107-75-5)	*Hydroxycitronellal*
Hydroxymethylpentylcyclohexenecarboxaldehyde (CAS 31906-04-4)	*Hydroxyisohexyl 3- cyclohexene-carboxaldehyde*
Isoeugenol (CAS 97-54-1)	*Isoeugenol*
Linalool (CAS 78-70-6)	*Linalool*
Methyl heptine carbonate (CAS 111-12-6)	*Methyl 2-octynoate*
Oak moss extract (CAS 90028-68-5)	*Evernia prunastri extract*
Treemoss extract (CAS 90028-67-4)	*Evernia furfuracea*
	*Extract*

Among the labels evaluated, as shown in Fig. 1, 371 (74%) had the term “perfume” in their composition. The term “flavoring,” used to designate substances with the property of intensifying or imparting aroma and/or flavor to foods,^6^ was found on 39 labels (7.8%), being more prevalent among toothpastes (97.4%).

**Figure 1:**
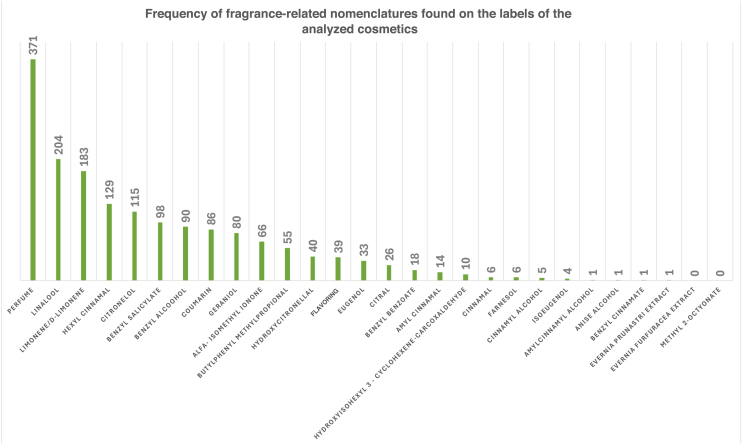
Frequency of terms found on the labels of the analyzed cosmetics.

In addition, it was observed that 91 products (18.2%) did not have any of the expressions “perfume,” “fragrance,” or “flavoring” on their labels, although they contained at least one of the 26 fragrances listed in ANVISA regulations. Among these substances, the following were identified: anise alcohol, benzyl benzoate, benzyl cinnamate, benzyl salicylate, cinnamyl alcohol, citral, citronellol, eugenol, isoeugenol, linalool, and hydroxycitronellal in one product each; geraniol and limonene/d-limonene in two products each; and benzyl alcohol in eight products. This finding is consistent with ANVISA RDC N. 83/2016, which requires individual labeling of these substances when present above established limits, but does not mandate the use of generic expressions such as “perfume” or “fragrance.” However, this approach may limit the consumer's perception of the presence of fragrances in the product, especially when associated with claims such as “organic,” “eco," “sensitive,” “pure,” or “natural,” which may induce a false sense of security or absence of sensitizing compounds. Corroborating this concern, a study conducted in the United States evaluated 187 products labeled as “hypoallergenic” and found that 89% of them contained at least one allergen, with fragrances being the second most prevalent group.^7^

Table 3 shows the frequency of the 26 fragrances evaluated according to the category of cosmetic products analyzed. The two most prevalent fragrances in the analyzed sample were linalool and limonene, present in all categories of cosmetics evaluated. Linalool is an essential oil extracted from plants such as tangerine, jasmine, lavender, bergamot, and coriander, and is frequently used in the industry as a fragrance fixative, in addition to having antimicrobial properties, among other applications.^8^ Limonene is used for its citrus scent, being the main constituent of oils derived from lemon and orange peels; in addition to cosmetics, it can be found in insect repellents.^9^ Both fragrances are most commonly used in combination in cleaning and cosmetic products. These presentations are considered pre-haptens and, therefore, incapable of causing contact dermatitis. However, when oxidized, they acquire a high sensitization potential, being common allergens in the series used in contact tests.^10^ Several similar studies, which analyzed the labels of cosmetics marketed in the European and Asian markets, also identified linalool and limonene as the most frequently present fragrance allergens in the formulations.^4^

**Table 3:** Frequency of the 26 fragrances evaluated according to the category of cosmetic products analyzed.

	Alcohol gel	Toothpastes	Sunscreen	Rinse-off hair care	Leave-on hair care	Hair dye	Rinse-off facial care	Leave-on facial care	Rinse-off body care	Leave-on body care
**Linalool**	9 (75%)	2 (5%)	11 (20%)	33 (52%)	23 (61%)	20 (39%)	9 (17%)	6 (13%)	44 (73%)	47 (55%)
**Limonene/** D-**limonene**	8 (67%)	22 (56%)	9 (16%)	20 (32%)	21 (55%)	11 (22%)	8 (15%)	6 (13%)	33 (55%)	45 (53%)
**Hexyl cinnamal**	5 (42%)	0	7 (13%)	34 (54%)	16 (42%)	7 (14%)	4 (8%)	3 (7%)	22 (37%)	31 (36%)
**Citronelol**	3 (25%)	0	8 (14%)	17 (27%)	9 (24%)	1 (2%)	6 (12%)	6 (13%)	27 (45%)	38 (45%)
**Benzyl salicylate**	2 (17%)	0	6 (11%)	19 (30%)	12 (32%)	10 (20%)	4 (8%)	3 (7%)	19 (32%)	23 (27%)
**Benzyl alcohol**	2 (17%)	17 (44%)	7 (13%)	15 (24%)	11 (29%)	9 (18%)	4 (8%)	7 (16%)	6 (10%)	12 (14%)
**Coumarin**	6 (50%)	0	2 (4%)	10 (16%)	10 (26%)	3 (6%)	1 (2%)	0	23 (38%)	31 (36%)
**Geraniol**	3 (25%	0	4 (7%)	8 (13%)	11 (29%)	2 (4%)	7 (13%)	2 (4%)	14 (23%)	29 (34%)
**Alfa-isomethyl ionone**	2 (17%)	0	5 (9%)	5 (8%)	8 (21%)	5 (10%)	1 (2%)	3 (7%)	10 (17%)	27 (32%)
**Butylphenyl methylpropional**	1 (8%)	0	4 (7%)	6 (10%)	3 (8%)	1 (2%)	6 (12%)	2 (4%)	16 (27%)	16 (19%)
**Hydroxycitronellal**	3 (25%)	0	5 (9%)	6 (10%)	5 (13%)	2 (4%)	2 (4%)	2 (4%)	3 (5%)	12 (14%)
**Eugenol**	0	5 (13%)	2 (4%)	0	0	0	0	2 (4%)	9 (15%)	8 (9%)
**Citral**	2 (17%)	2 (5%)	1 (2%)	2 (3%)	7 (18%)	0	0	1 (2%)	9 (15%)	9 (11%)
**Benzyl benzoate**	1 (8%)	0	3 (5%)	2 (3%)	2 (5%)	1 (2%)	1 (2%)	2 (4%)	1 (2%)	5 (6%)
**Amyl cinnamal**	1 (8%)	0	4 (7%)	2 (3%)	0	0	2 (4%)	0	2 (3%)	3 (4%)
**Hydroxyisohexyl 3 - cyclohexene-carcoxaldehyde**	0	0	2 (4%)	0	0	0	1 (2%)	1 (2%)	0	6 (7%)
**Cinnamal**	0	2 (5%)	0	1 (2%)	0	0	0	0	2 (3%)	1 (1%)
**Farnesol**	0	0	0	1 (2%)	0	0	0	1 (2%)	0	4 (5%)
**Cinnamyl alcohol**	0	0	0	0	0	0	0	1 (2%)	2 (3%)	2 (2%)
**Isoeugenol**	0	0	1 (2%)	0	0	0	0	1 (2%)	0	2 (2%)
**Amylcinnamyl alcohol**	0	0	0	0	0	0	0	0	1 (2%)	0
**Anise alcohol**	0	0	0	0	0	0	0	1 (2%)	0	0
**Benzyl cinnamate**	0	0	0	0	0	0	0	1 (2%)	0	0
**Evernia prunastri extract**	0	0	0	0	0	0	0	0	0	1 (1%)
**Evernia furfuracea extract**	0	0	0	0	0	0	0	0	0	0
**Methyl 2-octynoate**	0	0	0	0	0	0	0	0	0	0

On the other hand, *Evernia furfuracea* extract and Methyl 2- octynoate were not observed in any of the products evaluated, a fact also described in similar studies,^3^, ^4^ in which they represented less than 1% of the products investigated.

The category of products with the highest frequency of occurrence of the analyzed fragrances was that of “leave-on body care” products, including deodorants, antiperspirants, alcohols, moisturizers, emollients, perfumed lotions, scar reduction gels and talcum powders. These findings are consistent with previous publications, which also identified deodorants and perfumes as the products with the highest number of fragrances in their composition.^11^ In a Danish study, the most common sources of ACD to fragrances were deodorants (mainly in men), perfumed lotions and fine fragrances (more common in women), shampoos, liquid soaps, aftershave lotions and lipsticks.^12^

The results of the present study demonstrate that potentially sensitizing fragrances are widely present in cosmetics sold in the market, including those whose labeling omits the terms “perfume”, “fragrance” or “flavoring”. The discrepancy between the composition of the products and the clarity of the information on the labels compromises the consumer's right to information and hinders the proper management of conditions such as ACD. The high frequency of substances such as linalool and limonene, known to be associated with allergic reactions, reinforces the importance of vigilance regarding their presence, especially in products for continuous use, such as deodorants, moisturizers and body lotions. Furthermore, it is important to highlight that linalool and limonene are not included in the patch test by the Brazilian standard series, making diagnosis difficult in patients sensitized to these substances. These findings point to the need for greater rigor in cosmetic labeling and educational measures aimed at the general public and healthcare professionals.

## ORCID ID

Mariana de Figueiredo Silva Hafner: 0000-0001-8322-3856

Rosana Lazzarini: 0000-0002-4893-3593

## Research data availability

The entire dataset supporting the results of this study was published in this article.

## Financial support

None declared.

## Authors' contributions

Ana Carolina Rodrigues: Approval of the final version of the manuscript; drafting and editing of the manuscript; collection, analysis, and interpretation of data; critical review of the literature; critical review of the manuscript.

Mariana de Figueiredo Silva Hafner: Approval of the final version of the manuscript; drafting and editing of the manuscript; collection, analysis, and interpretation of data; effective participation in research orientation; critical review of the literature; critical review of the manuscript.

Rosana Lazzarini: Approval of the final version of the manuscript; design and planning of the study; drafting and editing of the manuscript; collection, analysis, and interpretation of data; effective participation in research orientation; critical review of the literature; critical review of the manuscript.

## Conflicts of interest

None declared.
